# Socio-economic dietary inequalities in UK adults: an updated picture of key food groups and nutrients from national surveillance data

**DOI:** 10.1017/S0007114514002621

**Published:** 2014-11-17

**Authors:** Eva R. Maguire, Pablo Monsivais

**Affiliations:** MRC Epidemiology Unit, UKCRC Centre for Diet and Activity Research (CEDAR), University of Cambridge School of Clinical Medicine, Institute of Metabolic Science, Box 285, Cambridge Biomedical Campus, CambridgeCB2 0QQ, UK

**Keywords:** Socio-economic differences, Diet, Inequalities, National Diet and Nutrition Survey, General linear models

## Abstract

Socio-economic differences in diet are a potential contributor to health inequalities. The present study provides an up-to-date picture of socio-economic differences in diet in the UK, focusing on the consumption of three food groups and two nutrients of public health concern: fruit and vegetables; red and processed meat; oily fish; saturated fats; non-milk extrinsic sugars (NMES). We analysed data for 1491 adults (age  ≥ 19 years) from the National Diet and Nutrition Survey 2008–2011. Socio-economic indicators were household income, occupational social class and highest educational qualification. Covariate-adjusted estimates for intakes of fruit and vegetables, red and processed meat, and both nutrients were estimated using general linear models. Covariate-adjusted OR for oily fish consumption were derived with logistic regression models. We observed consistent socio-economic gradients in the consumption of the three food groups as estimated by all the three indicators. Contrasting highest and lowest levels of each socio-economic indicator, we observed significant differences in intakes for the three food groups and NMES. Depending on the socio-economic indicator, highest socio-economic groups consumed up to 128 g/d more fruit and vegetables, 26 g/d less red and processed meat, and 2·6 % points less NMES (*P*< 0·05 for all). Relative to lowest socio-economic groups, highest socio-economic groups were 2·4 to 4·0 times more likely to eat oily fish. No significant patterns in saturated fat consumption were apparent. In conclusion, socio-economic differences were identified in the consumption of food groups and one nutrient of public health importance. Aligning dietary intakes with public health guidance may require interventions specifically designed to reduce health inequalities.

There are substantial socio-economic differences in the rates of obesity and chronic diseases, including type 2 diabetes and CVD^(^ ^1^ ^–^ ^6^ ^)^. Diet is a modifiable risk factor for such outcomes and, as such, is a likely contributor to health inequalities^(^ ^7^ ^,^ ^8^ ^)^. Understanding the social and economic patterning of diet is important for informing public health action, as recognised by Public Health England in 2013^(^ ^9^ ^)^.

Social gradients in diet have been identified in observational studies, with the majority of evidence on fruit and vegetable intake^(^ ^10^ ^–^ ^13^ ^)^. The association of diet with socio-economic position (SEP) has also been examined for the consumption of other food groups and nutrients including fish, processed meat and saturated fats^(^ ^10^ ^,^ ^12^ ^,^ ^14^ ^–^ ^18^ ^)^. Less healthful consumption has been consistently found among lower socio-economic groups, while compared with foods, nutrients have been less strongly associated with SEP^(^ ^12^ ^,^ ^19^ ^)^. However, existing studies have defined food and nutrient groups differently. For example, meat intake has been defined as the intake of processed meat^(^ ^16^ ^,^ ^20^ ^)^, fatty compared with lean meat^(^ ^14^ ^)^, and all meat and processed meat^(^ ^15^ ^)^, while social patterns of fat intake have been assessed as either total fat^(^ ^7^ ^,^ ^12^ ^)^ or saturated fat^(^ ^10^ ^,^ ^12^ ^)^ intake. This limits comparisons of intakes across socio-economic groups with dietary recommendations of public health concern, for which standardised definitions are necessary.

Understanding the socio-economic patterning of diet also requires full consideration of the material and social conditions characterising social stratification that may influence diet behaviours. Existing studies have employed one or more of the common indicators of SEP observed to represent such stratification: income; occupational social class; educational attainment^(^ ^21^ ^,^ ^22^ ^)^. Although related, the indicators are not interchangeable, with previous work identifying their independent contribution to dietary outcomes and emphasising the importance of using more than one indicator to fully characterise the socio-economic patterning of diet^(^ ^23^ ^,^ ^24^ ^)^. Few studies have used all the three indicators, despite some evidence that the extent of inequalities in diet differs depending on the indicator of SEP used^(^ ^24^ ^)^.

The aim of the present study was to estimate dietary inequalities in UK adults by three separate indicators of SEP in the consumption of food groups and nutrients of public health concern, using contemporary, nationally representative data and defining the food groups and nutrients according to national dietary guidelines. The present study examined the following issues: whether socio-economic gradients existed for all the selected food groups and nutrients; whether the nutrients were as strongly patterned as the food groups; whether any one indicator demonstrated stronger patterning than others.

## Methods

### Data source – the National Diet and Nutrition Survey 2008–2011

#### Sample

Data analysed in the present study were collected between 2008 and 2011 as part of the National Diet and Nutrition Survey (NDNS) rolling programme (years 1, 2 and 3). Briefly, the NDNS is a nationally representative sample of non-institutionalised residents of the UK aged 1·5 years and older, with age and sex weighting reflecting population distributions. Findings inform nutritional guidelines and are used to monitor the progress on dietary objectives set out by UK Health Departments^(^ ^25^ ^)^. The survey aimed to recruit 1000 participants per survey year, half adults (age  ≥ 19 years) and half children (age 1·5–18 years). Through random clustered sampling, 4595 households were selected, with up to one adult and one child randomly selected from each household. Response rates of fully productive individuals (those completing three or four dietary recording days) were 55 % for year 1, 55 % for year 2 and 52 % for year 3, giving a total sample of 3073 individuals^(^ ^26^ ^)^. Further details on sampling, data collection and processing are available elsewhere^(^ ^25^ ^,^ ^26^ ^)^. Adults were selected for the present analysis (age  ≥ 19 years, *n* 1491).

#### Sociodemographic information

Personal and household sociodemographic information of participants was collected during the face-to-face computer-assisted personal interview. The following available variables were included: age; sex; ethnicity (white, non-white); household size; household composition; highest educational qualification (eight categories); household income (thirteen categories); employment status; occupational social class (National Statistics Socio-Economic Classification, eight categories (NS-SEC8)); self-assessed general health. For the current purpose of the analysis, income and education were recoded.

##### Income

Total household income over the previous 12 months was equivalised to adjust for the presence of other adults and children in the household, using a rescaled version of the Organisation of Economic Development's modified equivalence scale^(^ ^27^ ^)^. This method of equivalisation was used previously in the analysis of the NDNS^(^ ^28^ ^)^. The midpoint of each category of household income was used to derive equivalised income, categorised into five income bands ( ≤ £14 999, £15 000–£24 999, £25 000–£34 999, £35 000–£49 999, £50 000 or more).

##### Education

For the present analysis, eight original categories for highest educational qualification were merged into six. All the six categories were included in statistical models, but estimates are only reported for the four categories considered ordinal: no qualifications; GCSE (General Certificate of Secondary Education)/equivalent; further or higher education below degree; degree or above. ‘GCSE and equivalent’ corresponds to academic school-leaving qualifications typically completed at 16 years of age or vocational courses of an equivalent level. ‘Further or higher education below degree’ represents academic and vocational courses that allow university entry (e.g. ‘A’ levels) or are to a foundation level in higher education; while ‘degree or above’ corresponds to the highest academic qualifications, including Bachelor's, Master's and Doctorate degrees. Estimates for the remaining two categories were not described as those ‘Still in full-time education’ had not yet obtained their highest qualification and the level of education obtained under ‘Foreign qualifications’ was unclear.

##### Occupational social class

The occupational social class of the survey household reference person is reported according to the NS-SEC8 (routine; semi-routine; lower supervisory and technical; small employers/own accounts; intermediate; lower managerial and professional; higher managerial and professional). Estimates for the eighth category, ‘never worked and long-term unemployed’, were excluded from the results as they would probably be unstable due to size (*n* 28) and incorporated those who did not work for reasons including long-term illness, which could confound dietary patterns. The NS-SEC has been in use in national statistics since 2001, and has a conceptual basis in employment relations and conditions of occupations, rather than distinguishing between levels of skill in employment^(^ ^29^ ^)^. Each NS-SEC class represents occupational groups with similar employment relations^(^ ^30^ ^)^. Although conceptually a nominal rather than an ordinal measure, the NS-SEC represents social class structure where behaviours and outcomes are expected to vary by class and within which certain classes are advantaged compared with others, for example those in higher managerial and professional roles have material advantage over intermediate employees or the routine, semi-routine and lower supervisory working classes^(^ ^30^ ^)^.

#### Dietary assessment

Dietary data were collected in the NDNS using estimated diaries over a consecutive 4 d period. The use of estimated diaries in the NDNS rolling programme was partly motivated by the need to reduce participant burden and minimise under-reporting compared with the weighed records that had been used previously^(^ ^31^ ^)^. The estimated diary method of dietary assessment has been previously validated against weighed dietary diaries^(^ ^32^ ^)^ and urinary markers of specific nutrients^(^ ^32^ ^,^ ^33^ ^)^. Although completed over four consecutive days, all days of the week were equally represented across the 3-year sample^(^ ^25^ ^)^. Weekend days were over-represented in the first study year, which were redressed in the second year^(^ ^26^ ^)^. Interviewers placed the diary with the participants and followed a protocol in explaining how to record food and drinks and portion sizes with an example day to illustrate^(^ ^31^ ^)^. Portion size estimation was guided by photographs of fifteen regularly consumed foods, household measures (e.g. tablespoons) and weights from labels (e.g. 420 g tins), with prompts to record leftover food from meals. Ingredients and their quantities in homemade dishes were recorded. Compliance to diary completion was aided by interviewers during and after the 4 d period^(^ ^26^ ^)^. Of the fully productive participants, 98 % completed four diary days^(^ ^26^ ^)^. Participants who did not complete three or four diary days were excluded from the NDNS (*n* 133)^(^ ^26^ ^)^, with no follow-up information provided for these participants. Under-reporting of energy intakes is a common issue in self-reported dietary methods^(^ ^34^ ^)^ and was expected in the sample^(^ ^26^ ^)^. However, we did not exclude on the basis of energy intakes as estimated energy requirements were not available for this sample.

Dietary data were processed using the DINO (Diet In Nutrients Out) database. Each recorded food or drink item was assigned a food code and a portion code linked to the corresponding weight of the item for the recorded portion. Coding of portions for adults was based on a reference from the Food Standards Agency (FSA), while weights for common branded foods and for foods from fast-food outlets were also available from the FSA^(^ ^31^ ^)^. Components of composite items (e.g. sandwiches) and homemade recipes were disaggregated to improve the estimates of the total amounts of individual foods consumed, particularly the meats, fish, fruit and vegetables food groups^(^ ^26^ ^)^. Nutrient intakes were calculated using the FSA's Nutrient Databank, with nutrient and energy values assigned to each food in the Databank^(^ ^26^ ^)^.

#### Food groups and nutrients of interest

We selected a range of food groups and nutrients reported in 2008 by the UK Scientific Advisory Committee on Nutrition (SACN) as consumed in unhealthful amounts in the population^(^ ^35^ ^)^, all of which have been included in previous analyses of SEP and diet. For the analysis, three food groups (fruit and vegetables, red and processed meat, and oily fish) and two nutrients (non-milk extrinsic sugars (NMES) and SFA) were selected *a priori* ^(^ ^35^ ^)^. The health bases for the dietary recommendations are displayed in Table 1. Fruit and vegetables and oily fish were consumed in insufficient amounts. A reduction in the intake of red and processed meat was recommended, with additional guidelines from the Department of Health in 2011 stating that those consuming 90 g/d are at an increased risk of colorectal cancer and should reduce their intake to 70 g/d^(^ ^36^ ^)^. SFA and NMES were consumed in excess of dietary reference values.

**Table 1:** Dietary recommendations for adults by the Scientific Advisory Committee on Nutrition^(^ ^35^ ^)^

Dietary factor	Recommendation	Reason for recommendation
Fruit and vegetables	Minimum 5 servings of 80 g/d (400 g)	To reduce the risk of CVD, some cancers, other chronic diseases
Red and processed meat	Individual consumption should not rise; high consumers should reduce consumption with the aim of reducing population average (90 g/d in 1998)	To reduce the risk of colorectal cancer
Oily fish	Minimum 140 g/week intake	To reduce the risk of CVD
Non-milk extrinsic sugars	Maximum 11 % food energy	To avoid dental caries
SFA	Maximum 11 % food energy	To reduce the risk of CVD; to reduce the energy density of diets

#### Dietary variables

Total energy intake calculated from the food diaries was available in the NDNS dataset. Intakes of the selected food groups and nutrients were presented in the NDNS dataset as follows: average daily intakes in grams of fruit and vegetables, red and processed meat, and oily fish; average percentage of daily food energy from NMES and SFA. The fruit and vegetables category included all fruits and vegetables in raw, cooked, frozen or canned form, including pulses and beans but excluding fruit juices and potatoes. All fruit and vegetable intakes from disaggregated recipes were included. Oily fish consisted of any oily fish or roe included in homemade dishes or any products containing oily fish such as canned fish, sushi or paste. Red and processed meat was aggregated from other relevant meat categories (including ‘beef’, ‘burgers’, ‘offal’ and ‘sausage’) and included fresh cuts, processed meats such as salami and sausage, meat consumed in homemade dishes, canned meat, and takeaway dishes^(^ ^37^ ^)^.

#### Ethical approval

The present study was a secondary analysis on the NDNS 2008–2011 data. The survey was conducted according to the guidelines established in the Declaration of Helsinki, and all procedures involving human subjects were approved by the Oxfordshire A Research Ethics Committee. The ethical declaration is also available in the study report^(^ ^26^ ^)^.

### Statistical analyses

Descriptive statistics were calculated for the adult sample. Unadjusted overall intakes were calculated to compare population intakes with nutritional recommendations and with trends reported by the SACN. Separate statistical models were built for each SEP indicator. Mean intake values and 95 % CI of fruit and vegetables, red and processed meat, NMES and SFA of socio-economic groups were obtained using general linear models. All models were adjusted for *a priori* identified covariates: age; sex; ethnicity; total energy intake; survey year. Models of red and processed meat consumption excluded non-consumers to reflect dietary guidelines, and non-consumers and consumers were compared using ANOVA and *t* tests.

Hypothesis tests were based on the differences between the highest and lowest SEP groups and the *F*-statistic testing for trend across the categories. Oily fish consumption was highly skewed with a large percentage of non-consumers, and so was converted to a binary outcome variable (0 = no consumption, 1 = any consumption) with OR derived from logistic regression models adjusted for age, sex, ethnicity, total energy intake and survey year. The lowest category for each socio-economic indicator was the referent for each analysis. Although not the focus of the present study, we also provided sex-specific estimates of food and nutrient intakes across the SEP groups and systematically tested for interactions between each SEP indicator and sex. Sex-specific estimates for socio-economic patterns of dietary intake are detailed in online supplementary Tables S1–S3.

Where results are reported in the text but not in tables, standard deviations are also reported. Individual-level weights were calculated by the NDNS to reduce the effect of potential sampling bias and differential non-response to participating by age, sex and region^(^ ^38^ ^)^. All analyses for the present study were accordingly weighted. All analyses were carried out in IBM SPSS version 20.0 (IBM Corp. 2011) and in Stata version 13.0 (StataCorp. 2013).

## Results

### Sample characteristics

The sample consisted of 1491 adults aged between 19 and 94 years, 51·4 % of which were women. The mean age for women was 49 (sd 18·3) years and for men 47 (sd 17·7) years. A quarter of men (26·4 %) and one-fifth of women (21·8 %) had attained a degree or higher, while more women than men had no qualifications (24·6 and 20·3 %, respectively). The full set of descriptive statistics is given in Table 2. Of the 1491 adults, 89 % of the sample (*n* 1350) reported consuming red and processed meat during the 4 d period (men 44·7 %, women 55·3 %). The majority of non-consumers were women (66·7 %). Compared with those included in the final analysis, non-consumers had significantly lower average energy intakes (*P*= 0·0002), with a difference of 799·7 (95 % CI 379·3, 1220·0) kJ/d. Non-consumers also differed in their socio-economic profiles, with significant differences in the distribution of participants across the income (*P*= 0·05), occupation (*P*= 0·0009) and education (*P*= 0·02) groups, suggesting that non-consumers of red and processed meat were of a lower SEP than consumers.

**Table 2:** Descriptive statistics of adult participants (age ≥19 years) of the National Diet and Nutrition Survey 2008–2011 (Number of adult participants and percentages, *n* 1491)

	Men	Women	Total	Missing data
	*n*	%	*n*	%	*n*	%	*n*	%
Total	724	48·6	767	51·4	1491	100	0	0
Age (years)							0	0
19–34	202	27·9	192	25·0	394	26·4		
35–50	228	31·4	235	30·6	463	31·0		
51–64	157	21·7	163	21·3	320	21·4		
65+	138	19·0	177	23·1	315	21·1		
Highest educational level							7	0·5
No qualifications	143	19·8	178	23·2	321	21·5		
GCSE/equivalent	156	21·5	161	21·0	317	21·3		
Further or higher education	177	24·4	189	24·7	366	24·6		
Degree or above	190	26·2	166	21·7	356	23·9		
Foreign qualification	32	4·4	34	4·4	66	4·4		
Full-time education	22	3·0	35	4·6	57	3·8		
Ethnicity							0	0
White	647	89·4	689	89·8	1336	89·6		
Non-white	77	10·6	78	10·2	155	10·4		
Equivalised household income (£) (12 months)							245	16·4
≤ 14 999	120	19·8	173	27·1	293	34·1		
15 000–24 999	147	24·2	151	23·6	298	25·6		
25 000–34 999	134	22·1	129	20·2	263	14·0		
35 000–49 999	105	17·3	96	15·0	201	7·5		
≥ 50 000	101	16·6	90	14·1	191	4·4		
Occupational class*							32	2·1
Routine	88	12·3	71	9·6	159	10·9		
Semi-routine	84	11·7	96	12·9	180	12·3		
Lower supervisory and technical	81	11·3	80	10·8	161	11·0		
Small employers	78	10·9	93	12·5	171	11·7		
Intermediate	48	6·7	78	10·5	126	8·6		
Lower managerial and professional	194	27·1	219	29·5	413	28·3		
Higher managerial and professional	133	18·6	88	11·8	221	15·1		
Never worked	10	1·4	18	2·4	28	1·9		

GCSE, General Certificate of Education.

* Occupational class of the household reference person.

### Overall intakes

The average daily energy intake was 8868 (sd 2654) kJ (2109 (sd 633·4) kcal) for men and 6680 (sd 1851) kJ (1588·2 (sd 441·0) kcal) for women. Adults consumed on average 290·8 (sd 171·7) g/d of fruit and vegetables, over 100 g short of the recommended daily amount. Respondents who ate red and processed meat consumed on average 78·0 (sd 51·2) g/d. Respondents consumed above dietary reference values of both nutrients, with on average 12·1 (sd 6·5) % of food energy constituted by NMES, and 13·0 (sd 3·5) % by SFA. A large proportion of participants ate no oily fish (72·2 %), while among those who did, average intake was low relative to the recommended levels (31·3 (sd 24·5) g/d).

### Adjusted average intakes by socio-economic position

Estimated average intakes of fruit and vegetables, red and processed meat, NMES and SFA across the socio-economic groups are summarised in Table 3.

**Table 3:** Adjusted* mean intakes (g/d) of the selected food groups and nutrients by socio-economic indicator (Mean values, percentage of food energy (% FE) and 95 % confidence intervals)

	FV	RPM	NMES	SFA
	Mean	95 % CI	Mean	95 % CI	% FE	95 % CI	% FE	95 % CI
Equivalised household income (£)†								
≤ 14 999	289·3	267·6, 310·9	76·3	69·6, 83·0	12·7	11·9, 13·5	12·2	11·8, 12·6
15 000–24 999	315·9	293·3, 338·6	71·7	64·7, 78·7	11·3	10·4, 12·1	12·6	12·1, 13·0
25 000–34 999	351·2	327·9, 374·6	71·1	63·9, 78·2	10·3	9·5, 11·2	12·4	11·9, 12·9
35 000–49 999	357·5	330·8, 384·2	63·6	55·6, 71·7	11·1	10·1, 12·1	12·2	11·7, 12·8
50 000+	386·4	359·8, 412·9	60·6	52·7, 68·6	10·1	9·1, 11·1	12·2	11·7, 12·8
*P* (highest and lowest group difference)	< 0·001	0·001	< 0·001	0·88
*P* (trend)	< 0·001	0·04	< 0·001	0·68
Occupational class‡								
Routine	253·9	226·9, 280·9	87·5	79·4, 95·6	12·3	11·3, 13·4	12·4	11·8, 12·9
Semi-routine	286·1	260·3, 311·8	76·7	68·8, 84·6	12·3	11·3, 13·3	12·2	11·7, 12·7
Lower supervisory and technical	316·6	289·2, 344·0	80·9	72·8, 89·1	11·1	10·1, 12·2	12·5	11·9, 13·0
Small employers/own accounts	329·1	302·5, 355·8	80·7	72·8, 88·6	10·9	9·9, 11·9	12·4	11·9, 13·0
Intermediate	305·2	274·3, 336·2	69·2	60·0, 78·4	11·3	10·1, 12·5	12·9	12·3, 13·5
Lower managerial and professional	340·6	321·6, 359·6	69·6	63·8, 75·4	11·3	10·6, 12·0	12·6	12·2, 13·0
Higher managerial and professional	367·6	343·4, 391·9	62·0	54·8, 69·3	10·8	9·9, 11·7	12·5	12·0, 13·0
*P* (highest and lowest group difference)	< 0·001	< 0·001	0·02	0·79
*P* (trend)	< 0·001	< 0·001	0·11	0·82
Highest educational level§								
No qualifications	256·5	234·6, 278·4	84·6	77·9, 91·4	12·0	11·1, 12·8	12·6	12·1, 13·0
GCSE/equivalent	293·0	271·7, 314·4	79·8	73·3, 86·3	12·2	11·3, 13·0	12·3	11·8, 12·7
Further or higher education below degree	316·6	296·5, 336·7	70·6	64·4, 76·9	11·5	10·7, 12·2	12·4	12·0, 12·8
Degree or higher	384·2	365·3, 403·0	62·7	56·8, 68·6	10·7	9·9, 11·4	12·3	11·9, 12·7
*P* (highest and lowest group difference)	< 0·001	< 0·001	0·01	0·31
*P* (trend)	< 0·001	< 0·001	0·06	0·11

FV, fruit and vegetables; RPM, red and processed meat; NMES, non-milk extrinsic sugars; GCSE, General Certificate of Education.

* Models adjusted for age, sex, ethnicity, total energy intake and survey year.

† Equivalisation based on the modified Organization for Economic Co-operation and Development (OECD) method described in the Methods section.

‡ Occupational class of the household reference person.

§ Highest educational level of the participant.

#### Fruit and vegetables

Average fruit and vegetable consumption was statistically significantly greater among the highest SEP participants compared with the lowest across the three indicators. The lowest-income participants consumed 97·1 g/d fewer fruit and vegetables than those with the highest incomes, with an increase in consumption across the income groups. The disparity between the most and least educated was 127·7 g/d, with an increase in intake alongside educational status, while there was a 113·7 g/d difference between those in routine occupations and those in higher managerial and professional occupations.

#### Red and processed meat

Among the adults who reported consuming any red and process meat, there was evidence of social gradients in intake, with a significant trend across each indicator. Participants in the lowest-earning households consumed 15·7 g/d more red and processed meat than the highest-earning households. Those with no qualifications consumed 21·9 g/d more red and processed meat than degree-educated participants. Participants in higher managerial and professional occupations consumed 25·5 g/d less red and processed meat than those in routine occupations.

#### Non-milk extrinsic sugars

NMES intake was negatively associated with income, with a significant difference of 2·5 % points of food energy between the lowest- and highest-income groups. Gradients were less consistent for occupation and educational attainment, with no significant trend across socio-economic levels (*P*= 0·108, 0·063, respectively). However, both of these SEP indicators showed a significant contrast between the lowest and highest groups. Those in routine occupations consumed significantly more NMES in the diet than those in the highest occupational group (12·3 *v.* 10·8 %, respectively) while those with no qualifications consumed significantly more NMES than the degree-educated group (12·0 *v.* 10·7 %, respectively).

#### SFA

There was no apparent patterning of SFA consumption for any of the socio-economic indicators.

#### Oily fish

Consumption of oily fish was patterned by all socio-economic indicators (see Table 4). All income groups had a significantly higher likelihood of consumption than the lowest group, with an OR of 4·0 for the highest-income participants and a gradient in odds across the income groups. Oily fish consumption increased by education level, with degree-educated participants having a nearly threefold increased likelihood than those with no qualifications. Only the two highest occupational groups were significantly more likely to consume oily fish than those in routine occupations.

**Table 4:** Adjusted† OR for oily fish consumption by socio-economic indicator (Adjusted odds ratios and 95 % confidence intervals)

	OR	95 % CI
Equivalised household income (£)‡		
≤ 14 999	Reference	
15 000–24 999	1·84*	1·21, 2·79
25 000–34 999	2·25**	1·47, 3·42
35 000–49 999	2·61**	1·67, 4·08
≥ 50 000	4·00**	2·58, 6·22
Highest educational level§		
No qualifications	Reference	
GCSE/equivalent	1·41	0·95, 2·09
Further or higher education below degree	2·03*	1·39, 2·97
Degree or higher	2·96*	2·01, 4·36
Occupational class∥		
Routine	Reference	
Semi-routine	0·95	0·55, 1·64
Lower supervisory and technical	0·85	0·48, 1·51
Small employers	1·42	0·84, 2·40
Intermediate	1·61	0·92, 2·82
Lower managerial and professional	2·25*	1·44, 3·51
Higher managerial and professional	2·43*	1·50, 3·93

GCSE, General Certificate of Secondary Education.

* Statistically significant at the 0·05 level.

** Statistically significant at the 0·01 level.

† Models adjusted for age, sex, ethnicity, total energy intake and survey year.

‡ Equivalisation based on the modified OECD method described in the Methods section.

§ Occupational class of the household reference person.

∥ Highest educational level of the participant.

### Socio-economic patterning by sex

Further analyses were conducted to test for socio-economic–diet interactions by sex and to provide sex-specific estimates of intakes for the three food groups and two nutrients. Across the socio-economic groups, fruit and vegetable intake was higher among women than among men, while red and processed meat was higher among men than among women. Sex interacted with occupation for red and processed meat intake and with education for red and processed meat intake, demonstrating heterogeneous gradients for these particular indicators and food groups. There were no significant interactions between any of the SEP indicators and sex for either NMES or SFA intakes. Oily fish consumption demonstrated stronger socio-economic gradients for women than for men. All the results are displayed in online supplementary Tables S1–S3.

## Discussion

A large existing evidence base has documented socio-economic inequalities in food consumption and nutrition in adults, with particular emphasis on fruit and vegetable intake^(^ ^10^ ^,^ ^11^ ^,^ ^13^ ^)^. The aim of the present study was to update the evidence base using nationally representative dietary data on UK adults, considering intakes of specific food groups and nutrients of public health concern and multiple indicators of SEP. The results in this sample aligned with SACN's 2008 report^(^ ^35^ ^)^ that dietary recommendations for the intakes of fruit and vegetables, oily fish, NMES and SFA are not met by the population. As expected, dietary shortfalls and excesses were unevenly distributed across the socio-economic groups.

Differentials were observed in the consumption of the three food groups examined and NMES by all the three socio-economic indicators, consistent with previous findings of greater fruit and vegetable intake^(^ ^10^ ^,^ ^13^ ^,^ ^14^ ^)^ and greater oily fish consumption^(^ ^16^ ^,^ ^18^ ^,^ ^39^ ^)^ among higher socio-economic groups. The socio-economic gradients identified for red and processed meat intake may be more pronounced between the consumption of lean, fresh red meat compared with processed meat, as more affluent groups have been found previously to consume more of the former and less of the latter^(^ ^14^ ^,^ ^16^ ^)^ with negative implications for health in consuming processed meat independent of red meat consumption^(^ ^40^ ^)^. While existing evidence for added sugar has shown a socio-economic gradient^(^ ^7^ ^,^ ^14^ ^)^, consistent socio-economic differences in SFA consumption have not been identified^(^ ^10^ ^,^ ^14^ ^)^, supporting earlier findings that nutrients are not as socially graded as food groups^(^ ^12^ ^,^ ^19^ ^)^.

Notably, no single SEP indicator demonstrated the strongest gradient for all foods and nutrients. One interpretation of this finding is that although pertaining to the same general concept of SEP, the indicators represent different aspects of social stratification, each with different mechanisms that influence dietary behaviours. For example, income reflects material resources to afford and access healthful foods^(^ ^24^ ^,^ ^41^ ^)^. The logic that lower income may limit the purchase of more costly, healthier foods is supported by evidence that diet cost is a probable mediator in the relationship between SEP and diet quality^(^ ^42^ ^)^. Further evidence of financial constraints on diet quality came from a recent report, which suggested that financial pressures from the recent economic recession and rising food prices had driven consumers to shift purchasing towards more energy-dense and processed foods and away from fruit and vegetables^(^ ^43^ ^)^. The differentials in intakes by income group identified in the present study may thus reflect these structural cost factors to an extent. In the case of occupational social class, the associated social environment can influence health behaviours through work-based culture and workplace social networks^(^ ^24^ ^,^ ^41^ ^)^, as social ties are likely to influence eating patterns^(^ ^44^ ^)^. Furthermore, recent work has demonstrated the associations between exposures to (unhealthful) takeaway food outlets in the work and commuting environments and dietary and health outcomes^(^ ^45^ ^)^, which themselves may be expected to vary by SEP. Therefore, there are social and environmental pathways through which occupational social class may influence particular dietary habits. Finally, a higher education level may pertain to increased competencies, skills and knowledge^(^ ^24^ ^,^ ^41^ ^)^, which are important for engaging with health education messages and avoiding harmful behaviours^(^ ^41^ ^)^. Education level is linked to dietary knowledge^(^ ^46^ ^,^ ^47^ ^)^, while a higher amount of education and knowledge can enable behaviours directed towards long-term benefits including healthier eating^(^ ^23^ ^)^. That there is not one indicator dominating the socio-economic gradients identified in the present study suggests that a range of such mechanisms may be at work to determine these differential intakes, and so further work on understanding these pathways in greater detail is required.

### Implications and further research

The selected food groups and nutrients analysed in the present study were listed by the SACN as important for health in their contribution to chronic disease rates. The present study replicated the SACN's definitions of the food groups, and compared intakes of both food groups and nutrients with the recommended levels. As such, future surveillance of dietary inequalities ought to employ the same categorisations in order to monitor socio-economic differences in diet. Although beyond the scope of the present study, further research could also characterise the full diet to provide insight into whether people are substituting between food groups and whether this differs by SEP.

In line with the selected food groups and nutrients, the broader trend of diets high in fat and sugar and low in fruits, vegetables, lean meat and fish is a burden on population health^(^ ^48^ ^)^, requiring continued efforts to alter it. Health promotion messages targeting nutritional shortfalls, such as the 5-A-Day fruit and vegetable campaign or the Food Standard Agency's 2009 saturated fat media campaign^(^ ^49^ ^,^ ^50^ ^)^, may need to be modified in order to more directly address dietary inequalities. Furthermore, the adoption of dietary guidance might have an associated financial cost. A recent analysis found that the diets of UK adults who met the 5-A-Day fruit and vegetable target were more costly than the diets of adults who failed to meet this target^(^ ^28^ ^)^. Fiscal incentives or other structural interventions may be appropriate to overcome financial barriers faced by households.

### Methodological considerations and limitations

The cross-sectional design of the study limits any causal inference between SEP and diet. With regard to NDNS data, the sample size contributed to uncertainty around subgroup estimates in the statistical models and limited our capacity to examine other population patterns. The survey response rate of over 50 % is a potential source of non-response bias, if those who participate are systematically different from those who do not. As the most deprived groups are less likely to participate in surveys^(^ ^51^ ^)^ the present results may under-represent those of the lowest SEP. Although measures were taken by the NDNS team to reduce the effect of potential non-response bias by calculating weights for the data^(^ ^38^ ^)^, the data may still contain bias. Finally, dietary data, including those presented here, are self-reported and so are subject to both random error and systematic error or bias^(^ ^34^ ^,^ ^52^ ^,^ ^53^ ^)^. In particular, energy intakes are known to be under-reported in dietary diaries^(^ ^34^ ^)^, while bias can arise from the misreporting of particular foods and products due to social desirability^(^ ^54^ ^)^. Although we adjusted our estimates for energy, which reduces the influence of this misreporting, and the measures taken by the NDNS to ensure complete recording of dietary intakes, there is still a chance that such biases may have led to over- or underestimated differences across the SEP groups, if the biases were socio-economically patterned.

Beyond these limitations, the strengths of the present study lie in its use of an up-to-date, nationally representative surveillance dataset to assess differences in the consumption of certain food groups and nutrients that potentially contribute to health inequalities among adults in the UK. The use of multiple SEP indicators allows the consideration of different dimensions of SEP, rather than considering SEP as a single phenomenon.

### Conclusions

The present study updates the picture of socio-economic inequalities in diet among UK adults in relation to specific food groups and nutrients of public health concern. Given the health concerns associated with either the under- or overconsumption of these food groups and nutrients, it is important to continue the surveillance of inequalities in diet, as there are implications for related health inequalities. National data sources such as the NDNS are appropriate to monitor such inequalities and ought to be utilised to this end. In the general adult population, dietary outcomes need to improve in order to meet dietary guidelines. As supported by the findings from the present study, dietary inequalities require additional attention, with any action to improve dietary behaviours at the population level also targeted at closing the socio-economic gap. Given the complexity of the factors associated with SEP, this is likely to require a range of strategies targeting psychosocial, behavioural and structural barriers to healthy eating.

## Supplementary material

To view supplementary material for this article, please visit http://dx.doi.org/10.1017/S0007114514002621
